# Supplementary material for: Shared genetic architecture of cortical morphology and psychiatric disorders: insights from a cross-trait analyses across 180 cortical regions
Source: medRxiv. 2026 Apr 13:2026.04.10.26349224. Preprint. [Version 1] doi: 10.64898/2026.04.10.26349224 (PMC13127536; doi:10.64898/2026.04.10.26349224)
Supplement: Supplement 1 [file media-1.pdf]

Shared genetic architecture of cortical morphology and psychiatric disorders: insights from a cross-trait analyses across 180 cortical regions

Yingzhe Zhang[1-4] PhD, Tian Ge[2,3,4] PhD, Travis T. Mallard[2,3,4] PhD, Karmel W Choi[2,3,4] PhD, Anxiety Disorders Working Group of the Psychiatric Genomics Consortium, Henning Tiemeier[1] MD, PhD, Sander Lamballais[5] PhD

1 Harvard T.H. Chan School of Public Health, Boston.

2 Psychiatric and Neurodevelopmental Genetics Unit, Center for Genomic Medicine, Massachusetts General Hospital, Boston.

3 Center for Precision Psychiatry, Department of Psychiatry, Massachusetts General Hospital, Boston.

4 Department of Psychiatry, Harvard Medical School, Boston,

5 Department of Clinical Genetics, Erasmus MC, University Medical Center Rotterdam, Rotterdam, Netherlands

Corresponding author:

Yingzhe Zhang PhD, Department of Epidemiology, Harvard T.H. Chan School of Public Health, 677 Huntington Ave, Boston, MA 02115 (yzhang1@hsph.harvard.edu)

Henning Tiemeier MD, PhD, Department of Social and Behavioral Sciences, Harvard T.H. Chan School of Public Health, 677 Huntington Ave, Kresge Building, Room 619, Boston, MA 02115 (tiemeier@hsph.harvard.edu)

Supplemental Figure 1 Distribution of genetic variants shared by a certain number of brain regions (cortical thickness and area) with psychiatric traits: Different patterns for specific psychiatric disorders, psychiatric disorder groups, and general psychopathology.

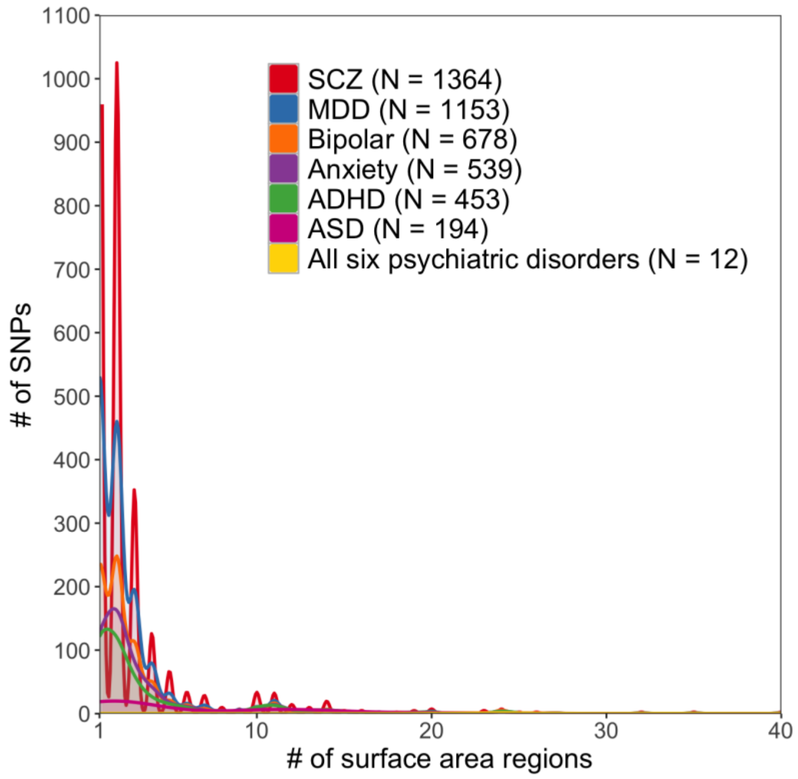

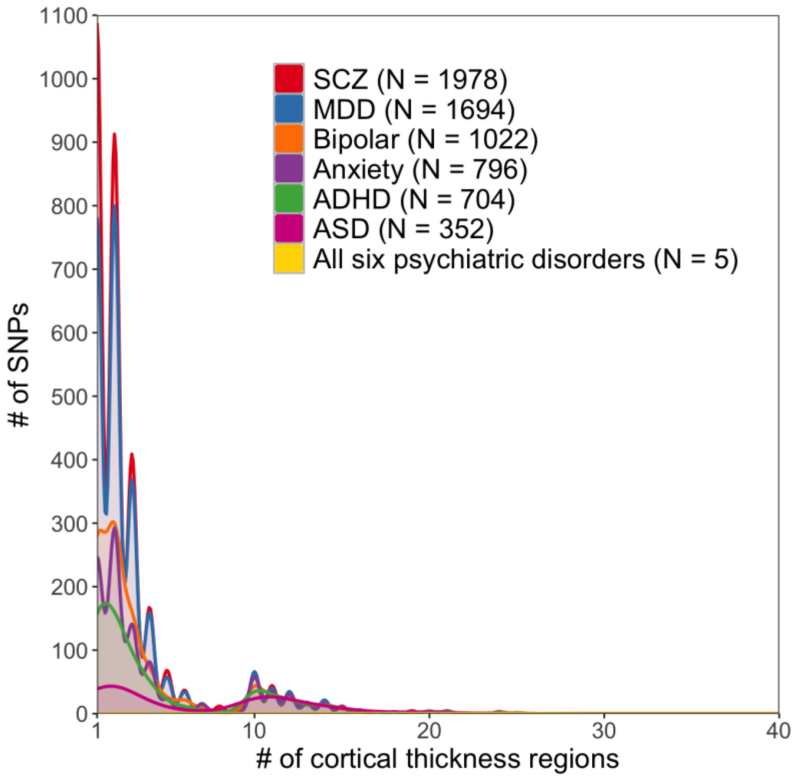

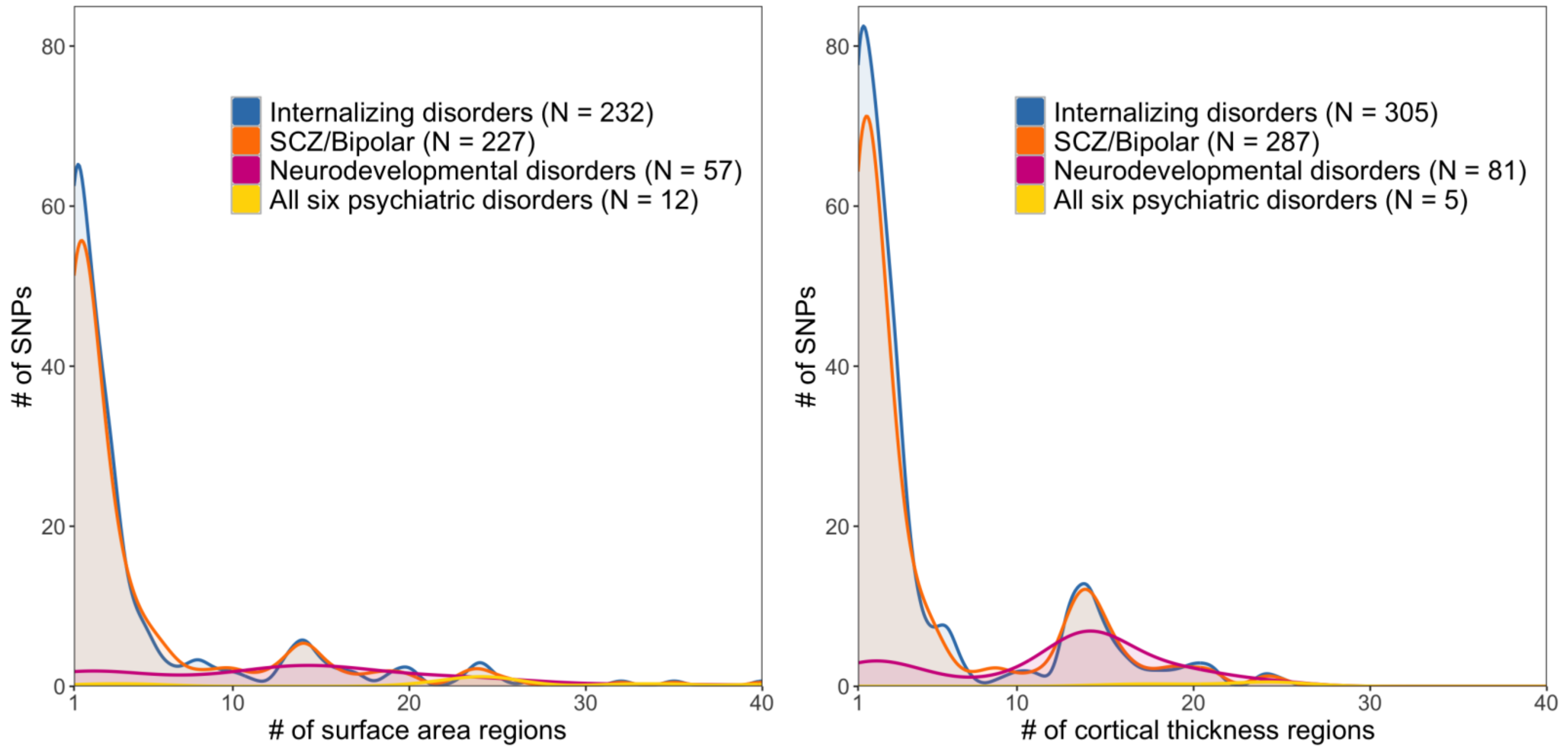
Internalizing disorders (N = 232)
SCZ/Bipolar (N = 227)
Neurodevelopmental disorders (N = 57)
All six psychiatric disorders (N = 12)
# of SNPs
# of surface area regions
Internalizing disorders (N = 305)
SCZ/Bipolar (N = 287)
Neurodevelopmental disorders (N = 81)
All six psychiatric disorders (N = 5)
# of SNPs
# of cortical thickness regions
